# Influence of different storage times and temperatures on blood gas and acid-base balance in ovine venous blood

**Published:** 2013-01-03

**Authors:** H.A. Hussein, A.A. Aamer

**Affiliations:** *Department of Animal Medicine, Faculty of Veterinary Medicine, Assiut University, Assiut 71526, Egypt*

**Keywords:** Acid-base indices, Clinical relevance, Ovine, Storage temperature time

## Abstract

The present study was designed to investigate the effects of storage temperature and time on blood gas and acid-base balance of ovine venous blood. Ten clinically healthy sheep were used in this study. A total number of 30 blood samples, were divided into three different groups, and were stored in a refrigerator adjusted to +4 ºC (Group I, *n* = 10), at RT of about 22-25 ºC (Group II, *n* = 10) and in an incubator adjusted to 37 ºC (Group III, *n* = 10) for up to 48 h. Blood samples were analysed for blood gas and acid-base indices at 0, 1, 2, 3, 4, 5, 6, 12, 24 and 48 h of storage. In comparison to the baseline value (0), there were significant decreases of blood pH of samples stored at RT and in the incubator after 1 h (*p*<0.05), the pH value of refrigerated blood samples exhibited insignificant changes during the study (*p*<0.05). Mean values of pCO_2_ showed a significant increase in Group I and Group III after 1 h then a progressive decrease after 12 h in all Groups. Mean pO_2_ values were significantly higher for Group I after 2 h and for Groups II and III after 1 h (*p*<0.05). In general, base excess decreased significantly for all the groups during the study especially in Groups II and III. In comparison with baseline values, in all groups, bicarbonate (HCO_3_) increased between 1 h and 6 h (*p*<0.05), and later decreased at the end of the study (*p*<0.05). In conclusion, status of acid-base indices of the samples stored at refrigerator and RT were found within normal reference range and it may be of clinical diagnostic use for up to 6 h.

## Introduction

Evaluations of blood gas and acid-base balance are an important tool in the diagnosis and treatment of diseases that affect sheep. Various metabolic and respiratory diseases affect venous blood gas composition and acid–base values of sheep (Carlson, 1996; Radostits *et al.*, 2006). Therefore, accurate estimation of these blood indices is of great importance for veterinary clinicians and research workers.

Accurate acid-base analysis requires careful sample management. For carrying out blood gas and acid-base analysis, blood samples should be collected anaerobically from a large, free-following vessel into a heparinised tube (George, 1986). In man (Knowles *et al.*, 2006), cattle (Gokce *et al.*, 2004) and goat (Leal *et al.*, 2010) blood gas composition and acid–base values may change depending on the type of syringe used for sampling, delays in measurement time and variations in storage temperature.

Continuous aerobic and anaerobic metabolism between the drawing of the blood samples and their analysis intervals may affect blood gas and acid-base indices in humans (Liss and Payne, 1993), cattle (Szenci and Besser, 1990; Gokce *et al.*, 2004), goat (Leal *et al.*, 2010) and dogs (Haskins, 1977). *In vitro*, blood metabolism includes both aerobic metabolism with production of carbon dioxide (CO_2_) and anaerobic glycolysis with production of lactic acid. Subsequent rising of partial tension of carbon dioxide (pCO_2_) is a common sequel for these metabolic activities in the stored blood (Haskins, 1977). Oxygen consumption is altered depending on the enzymatic activities of the citric acid and cytochrome systems in leucocytes and reticulocytes. Furthermore, glycolysis is the predominant metabolic activity of mature erythrocytes (Szenci and Besser, 1990).

As previously demonstrated for measurement of blood gas and acid-base indices, blood samples must be measured immediately if kept at room temperature (RT), or within 3 hours if kept on ice (+4ºC) (George, 1986; Duncan and Prasse, 1986). Under field condition, it is difficult to estimate the blood gas and acid-base variables in a short period of time; therefore it may be necessary to store blood samples until analysis (Szenci and Besser, 1990).

Several studies have investigated the changes in blood gas composition and acid-base values during storage of blood samples from cattle (Szenci and Besser, 1990; Gokce *et al.*, 2004), goat (Leal *et al.*, 2010) and dogs (Haskins, 1977).

It is very important to examine the stability of blood gas and acid-base indices in stored ovine blood, as there is a lack of consensus in literature regarding the extends of changes in blood gas and acid-base values that may affect the clinical interpretation of ovine venous blood stored at (RT) (22-25 ºC) or at 37 ºC for long period of time. Therefore, this study was designed to determine the influence of storage of blood samples for up to 48 h at different temperatures (+4, 22-25 and 37 ºC) on blood gas composition and acid-base indices in ovine venous blood.

## Materials and Methods

### Animals and sampling

Ten clinically healthy, 4-6 year old, Baladi non-pregnant ewes were used in this study. All animals were under the same feeding and management systems. On the day of sampling, rectal temperature of each animal was measured as well as faecal samples were collected. Anaerobically, three jugular venous blood samples were drawn from each animal into 3 ml plastic syringes (containing freeze-dried lithium heparin with a 23 gauge × 1-inch needle). Immediately after venipuncture, the tip of the needle was sealed with a rubber stopper in order to prevent gas from moving in or out of it (Haskins, 1977).

The samples were placed in a bed of crushed ice, taken immediately to the laboratory and analysed within 10 min. Following the first (0) hour’s analysis, a total 30 blood samples were allocated into three groups and then stored, respectively, in a refrigerator set to +4 ºC (Group I, *n* = 10), at RT ranging from 22 to 25 ºC (Group II, *n* = 10) and in an incubator adjusted to 37 ºC (Group III, *n* = 10), for up to 48 h. Blood gas and acid/base indices were analysed at 0, 1, 2, 3, 4, 5, 6, 12, 24 and 48 h of storage for all groups. For haematological examinations, blood samples were collected from the jugular vein into evacuated ethylenediaminetetraacetic acid (EDTA) containing tubes.

### Clinical examination

All animals were examined clinically with measurements of rectal temperature, pulse rate, respiratory rate, and ruminal cycle as described by Pugh (2002).

### Acid-base and blood gas analysis

The blood samples were analysed for pH, pCO_2_, partial tension of oxygen (pO_2_), actual base excess (BEact), standard base excess (BEstd), standard bicarbonate (stdHCO_3_), actual bicarbonate (actHCO_3_) concentrations, total carbon dioxide content (tCO_2_) and oxygen saturation (O_2_SAT) using blood gas analyser (ABL 5, Radiometer, Copenhagen, Denmark).

### Haematological analysis

Blood samples collected in EDTA were used for estimation of total red blood cells (RBCs), haemoglobin concentration (Hb) and total white blood cells (WBCs), manually as described by Coles (1980).

### Statistical methods

Values are presented as the means ± standard error of the mean (SE). For statistical evaluations, all data were processed via the Statistical Package for Social Science (version 10.0 for Windows).

Blood analytics were subjected to a two-way analysis of variance (ANOVA) with main effects of storage temperature (group), time intervals, and their interaction. The statistical significance of differences between group means was estimated by post hoc analysis using the least significant difference (LSD) test at *p*<0.05 (Dunn and Clark, 2009).

## Results

The mean values of clinical parameters of the animals used in this study were detected as 38.6 ± 0.06 ºC for rectal temperature, 80 ± 0.91 beats/min for pulse rate, 23 ± 0.42 breaths/min for respiratory rate and 2.1 cycles/2 min for ruminal cycle. The mean initial haematological values of sheep studied were detected as 7.0 ± 0.3 T/l for RBCs, 110 ± 2.0 g/l for Hb and 7.7 ±0.6 G/l for WBCs. Faecal analysis revealed no internal parasitic infection.

**Fig. 1:**
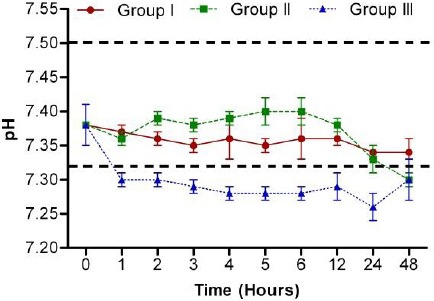
Influence of storage time on the pH of ovine blood samples stored at +4 ºC (Group I), 22-25 ºC (Group II) and 37 ºC (Group III) (*n* = 10) (mean ± SE). Dashed horizontal lines represent the reference values (pH = 7.32 – 7.5) according to Kaneko *et al*. (2008).

Blood gas and acid-base indices were summarised in tables 1-4 and Fig. 1. The pH values of the samples stored at 22-25 ºC and 37 ºC tended to be altered significantly with time (Table 3). In comparison with the initial values, pH values increased significantly after 2 h, for samples stored at 22-25 ºC (*p*<0.05), while pH values decreased significantly after 1 h for samples stored at 37 ºC (*p*<0.05).

**Table 1:** Average values of pCO_2_ and pO_2_ measured at regular time intervals in ovine venous blood samples stored at +4 ºC (Group I), 22-25 ºC (Group II) and 37 ºC (Group III) (n = 10) (mean ± SE)

parameter	Group	Time (hours)										
		0	1	2	3	4	5	6	12	24	48	P1
pCO_2_ (mmHg)	I	42±0.7	52±3.5	49±2.3	49±2.3	47±2.0	47±2.1	47±2.0	33±2.0	20±2.0	8±1.0	*p*<0.001
		b	a	AB,a	A,a	AB,ab	A,ab	A,ab	c	d	C,e	
	II	42±0.7	46±2.8	42±2.5	42±2.3	41±2.4	39±2.4	39±2.4	28±2.5	25±3.9	21±1.9	*p*<0.001
		a	a	B,a	B,a	B,a	B,a	B,a	b	bc	A,c	
	III	42±0.7	56±2.6	52±2.3	A,52±2.0	52±2.1	52±2.1	49±1.5	33±2.7	22±2.6	15±0.3	*p*<0.001
		c	a	A,ab	A,ab	A,ab	A,ab	A,b	d	e	B,f	
P2				*p*<0.05	*p*<0.01	*p*<0.01	*p*<0.001	*p*<0.01			*p*<0.001	
pO_2_ (mmHg)	I	36±1.0	41±0.6	42±0.7	44±1.0	47±0.6	47±1.0	50±0.7	53±0.8	60±1.0	75±6.0	*p*<0.001
		g	B,fg	B,ef	B,ef	B,de	B,de	B,cd	B,c	B,b	A,a	
	II	36±1.0	51±2.2	53±2.3	55±2.6	58±2.4	61±2.7	61±3.1	66±3.1	68±4.5	65±6.3	*p*<0.001
		f	A,e	A,ed	A,ed	A,bcde	A,abcd	A,abcd	A,abc	A,a	A,ac	
	III	36±1.0	39±0.9	40±0.7	40±0.7	41±0.9	41±1.2	40±1.3	37±2.4	25±2.2	24±0.4	*p*<0.001
		d	B,abc	B,ab	B,ab	C,a	C,a	C,a	C,cbd	C,e	B,e	
P2			*P*<0.001	*p*<0.001	*p*<0.001	*p*<0.001	*p*<0.001	*p*<0.001	*p*<0.001	*p*<0.001	*p*<0.001	

P1, significant differences within group compared to baseline value (a,b,c,d,e,f,g); P2, in each column different letters (A, B, C) indicated significant between group (*p*< 0.05), (*p*< 0.01), (*p*< 0.001); pCO_2_, partial pressure of carbon dioxide (mmHg); pO_2_, partial pressure of oxygen (mmHg).

**Table 2:** Average values of tCO_2_ and O_2_ SAT measured at regular time intervals in ovine venous blood samples stored at +4 ºC (Group I), 22-25 ºC (Group II) and 37 ºC (Group III) (*n* = 10) (mean ± SE)

parameter	Group	Time (hours)										
		0	1	2	3	4	5	6	12	24	48	P1
tCO_2_ (mmol/l)	I	21±0.5	29±1.2	27±0.8	27±0.8	27±0.7	27±0.8	26±0.8	18±0.7	11±0.9	5±0.3	*p*<0.001
		c	a	ab	ab	ab	ab	b	d	f	B,e	
	II	21±0.5	27±0.9	26±0.8	26±0.8	25±0.8	24±0.9	25±0.9	17±1.0	13±1.6	8±1.3	*p*<0.001
		b	a	a	a	a	a	a	c	d	A,e	
	III	21±0.5	28±0.9	26±0.7	26±0.7	26±0.6	25±0.6	25±0.6	16±0.9	10±0.9	10±0.5	*p*<0.001
		c	a	b	b	b	b	b	d	e	A,e	
P2												*p*<0.001	
O_2_ SAT (%)	I	80±3.5	72±1.5	76±2.2	76±1.6	80±0.6	80±1.5	83±0.7	86±0.4	90±0.6	94±0.8	*p*<0.001
		cd	B,e	B,de	B,de	B,cd	B,cd	B,cb	A,bf	A,af	A,a	
	II	80±3.5	83±2.0	86±1.4	86±1.8	89±1.1	88±1.3	90±1.2	92±1.0	92±1.1	89±1.6	*p*<0.001
		f	A,df	A,bcd	A,bcd	A,ac	A,ac	A,ac	A,a	A,a	B,ac	
	III	80±3.5	68±1.4	68±1.3	68±1.4	71±1.2	69±2.3	69±2.4	62±5.4	42±5.0	32±0.4	*p*<0.001
		a	B,bc	C,bc	C,bc	C,bd	C,bc	C,bc	B,c	B,e	C,f	
P2			*p*<0.001	*p*<0.001	*p*<0.001	*p*<0.001	*p*<0.001	*p*<0.001	*p*<0.001	*p*<0.05	*p*<0.001	

P1, significant differences within group compared to baseline value (a,b,c,d,e,f); P2, in each column different letters (A, B, C) indicated significant between group (*p*<0.05), (*p*<0.001); tCO_2_, total carbon dioxide (mmol/l); O_2_ SAT, oxyhaemoglobin saturation (%).

**Table 3:** Average values of pH, BEact and BEstd measured at regular time intervals in ovine venous blood samples stored at +4 ºC (Group I), 22-25 ºC (Group II) and 37 ºC (Group III) (*n* = 10) (mean ± SE)

parameter	Group	Time (hours)										
		0	1	2	3	4	5	6	12	24	48	P1
pH	I	7.38 ±0.03	7.37 ±0.01	7.36 ±0.01	7.35 ±0.01	7.36 ±0.03	7.35 ±0.01	7.36 ±0.03	7.36 ±0.01	7.34 ±0.01	7.34 ±0.02	*P*=0.389
		a	A,a	B,a	B,a	B,a	B,a	B,a	A,a	A,a	a	
	II	7.38 ±0.03	7.36 ±0.01	7.39 ±0.01	7.38 ±0.01	7.39 ±0.01	7.40 ±0.02	7.40 ±0.02	7.38 ±0.01	7.33 ±0.02	7.30 ±0.01	*p*<0.01
		ab	A,bc	A,a	A,a	A,a	A,a	A,a	A,a	A,c	d	
	III	7.38 ±0.03	7.30 ±0.01	7.30 ±0.01	7.29 ±0.01	7.28 ±0.01	7.28 ±0.01	7.28 ±0.01	7.29 ±0.02	7.26 ±0.02	7.30 ±0.03	*p*<0.001
		a	B,b	C,b	C,bc	C,bc	C,bc	C,bc	B,bcd	B,cd	bc	
P2				*p*<0.01	*p*<0.001	*p*<0.001	*P*<0.001	*p*<0.001	*p*<0.001	*p*<0.001	*p*<0.01		
BEact (mmol/l)	I	0.40 ±0.4	1.20 ±0.6	0.60 ±0.3	-0.20 ±0.4	0.00 ±0.4	-0.20 ±0.4	-0.20 ±0.4	-6.80 ±0.6	-13.20 ±0.8	-21.60 ±0.7	*p*<0.001
		a	a	A,a	A,a	A,a	A,a	A,a	A,b	A,c	B,d	
	II	0.40 ±0.4	0.00 ±0.5	-0.20 ±0.3	-0.20 ±0.3	-0.20 ±0.3	-0.80 ±0.5	-0.80 ±0.5	-7.40 ±0.6	-11.80 ±1.0	-17.60 ±1.1	*p*<0.001
		a	a	A,a	AB,a	A,a	A,a	A,a	A,b	A,c	A,d	
	III	0.40 ±0.4	-0.60 ±0.6	-2.00 ±0.7	-2.60 ±0.5	-2.80 ±0.5	-3.60 ±0.4	-3.60 ±0.4	-10.20 ±0.6	-16.40 ±0.7	-22.60 ±0.3	*p*<0.001
		a	ab	B,bc	B,cd	B,cd	B,d	B,d	B,e	B,f	B,g	
P2					*p*<0.01	*p*<0.01	*P*<0.001	*p*<0.001	*p*<0.001	*p*<0.001	*p*<0.05	*p*<0.001	
BEstd (mmol/l)	I	0.60 ±0.3	2.40 ±0.9	0.80 ±0.5	1.00 ±0.6	0.40 ±0.5	0.40 ±0.5	0.40 ±0.5	-7.20 ±0.7	-14.20 ±0.8	-22.80 ±0.6	*p*<0.001
		b	a	ab	A,ab	A,b	A,b	A,b	A,c	AB,d	B,e	
	II	0.60 ±0.3	0.20 ±0.6	0.40 ±0.6	0.00 ±0.6	-0.60 ±0.6	-1.00 ±0.7	-0.60 ±0.6	-8.20 ±0.9	-12.40 ±1.3	-18.0 ±1.3	*p*<0.001
		a	a	a	AB,a	AB,a	AB,a	A,a	AB,b	A,c	A,d	
	III	0.60 ±0.3	1.00 ±0.7	-0.80 ±0.7	-1.20 ±0.6	-2.00 ±0.5	-2.40 ±0.6	-2.60 ±0.6	-10.40 ±0.7	-16.80 ±0.9	-23.40 ±0.3	*p*<0.001
		ab	a	bc	B,cd	B,cd	B,bcd	B,d	B,e	B,f	B,g	
P2					*p*<0.05	*p*<0.05	*p*<0.01	*p*<0.01	*p*<0.05	*p*<0.01	*p*<0.001	

P1, significant differences within group compared to baseline value (a,b,c,d,e,f,g); P2, in each column different letters (A, B, C) indicated significant between group (*p*<0.05), (*p*<0.01), (*p*<0.001); BEact, extracellular base excess (mmol/l); BEstd, blood base excess, (mmol/l).

**Table 4:** Average values of ActHCO_3_ and StdHCO_3_ measured at regular time intervals in ovine venous blood samples stored at +4 ºC (Group I), 22-25 ºC (Group II) and 37 ºC (Group III) (*n* = 10) (mean ± SE)

parameter	Group	Time (hours)										
		0	1	2	3	4	5	6	12	24	48	P1
ActHCO_3_ (mmol/l)	I	21±0.3	28±1.1	26±0.7	26±0.7	25±0.6	25±0.6	25±0.7	17±0.8	10±0.9	4.4±0.4	*p*<0.001
		c	a	b	b	b	b	b	d	AB,e	B,f	
	II	21±0.3	25±0.8	25±0.7	24±0.7	24±0.8	23±0.8	23±0.8	16±1.0	13±1.5	8±1.2	*p*<0.001
		b	a	a	a	a	ab	ab	d	A,e	A,f	
	III	21±0.3	27±0.8	25±0.6	24±0.6	24±0.6	23±0.6	23±0.6	15±0.9	9±0.9	8±0.3	*p*<0.001
		c	a	ab	b	b	bc	bc	d	B,e	A,e	
P2											*p*<0.05	*p*<0.01	
StdHCO_3_ (mmol/l)	I	20±0.3	25±0.5	24±0.8	24±0.4	24±0.4	24±0.4	24±0.5	19±0.5	14±0.4	9±0.6	*p*<0.001
		b	a	A,a	A,a	A,a	A,a	A,a	A,c	A,d	B,e	
	II	20±0.3	24±0.5	24±0.3	24±0.3	24±0.3	24±0.4	24±0.4	18±0.4	15±0.8	12±0.6	*p*<0.001
		b	a	A,a	A,a	A,a	A,a	A,a	A,c	A,d	A,e	
	III	20±0.3	23±0.5	22±0.5	22±0.5	21±0.4	21±0.4	21±0.4	16±0.4	12±0.5	10±0.3	*p*<0.001
		cd	a	B,b	B,b	B,cb	B,cb	B,c	B,e	B,f	B,g	
P2				*p*<0.01	*p*<0.001	*p*<0.001	*p*<0.001	*p*<0.001	*p*<0.001	*p*<0.001	*p*<0.001	

P1, significant differences within group compared to baseline value (a,b,c,d,e,f); P2, in each column different letters (A, B, C) indicated significant between group (*p*<0.05), (*p*<0.01), (*p*<0.001); ActHCO_3_, actual bicarbonate concentrations (mmol/l); StdHCO_3_, standard bicarbonate concentration (mmol/l).

Although the values of pH fluctuated with time in groups I and II, it remained within the normal reference range especially between 1 h and 12 h. Moreover, in Group III, pH values were lower than the lower reference limit (Fig. 1).

Changes in pCO_2_ and pO_2_ are summarised in Table 1. Between 1 and 6 h, the mean pCO_2_ values of samples stored at +4 and 37 ºC increased significantly (*p*<0.05), while they remained nearly without noticeable changes at 22-25 ºC.

In all groups, mean values of pCO_2_ decreased to values lower than their initial values after 6 h (*p*<0.05). The mean values of pO_2_ of samples kept at +4 and 22-25 ºC increased with time (*p*<0.001). However, a significant decrease was recorded after 24 h for samples stored at 37 ºC (*p*<0.05).

Alterations in tCO_2_ and O_2_ SAT values are summarised in Table 2. In all groups, the mean tCO_2_ values increased significantly between 1 and 6 h (*p*<0.05), but later decreased to values lower than their initial values (*p*<0.05). Values of O_2_ SAT in Group I decreased after 1 h (*p*<0.05) then increased gradually until the end of the study. On the other hand O_2_ SAT values significantly increased in Group II (*p*<0.001), but they significantly decreased in Group III (*p*<0.001) until the end of the study.

Base excess concentrations nearly decreased for all three groups during the study compared to their baseline values except in Group I, BEstd concentration increased after 1 h (*p*<0.05) then decreased thereafter. The most prominent decrease in BE values was observed in samples stored at 37 ºC (Table 3). In comparison with baseline values, ActHCO_3_ and StdHCO_3_ values increased in samples kept at +4 ºCbetween 1 and 6 h (*p*<0.05), later decreasing to values lower than their base values (*p*<0.05). In comparison with baseline values of Group II, ActHCO_3_ increased until 4 h (*p*<0.05) then significantly decreased (*p*<0.05), while StdHCO_3_ increased until 6 h (*p*<0.05) then significantly decreased (*p*<0.05). In Group III, values of ActHCO_3_ increased between 1 and 4 h then gradually decreased thereafter when compared with the initial value (*p*<0.05), while values of StdHCO_3_ increased between 1 and 3 h then decreased until the end of study (*p*<0.05) (Table 4). There were significant time-group (storage temperature) interactions on all measured parameters (Table 5).

**Table 5:** Influence of time, storage temperature (group of animals), and their interaction on blood gases and acid-base balance of ovine venous blood (*n* = 30)

Parameters	Main effects (*P < F*)		
	Time	Group	Time × Group
pH	0.228	<0.001	0.001
pCO_2_ (mmHg)	<0.001	0.011	<0.001
pO_2_ (mmHg)	0.869	<0.001	<0.001
ActHCO_3_ (mmol/l)	<0.001	0.374	0.002
StdHCO_3_ (mmol/l)	<0.001	<0.001	<0.001
tCO_2_ (mmol/l)	<0.001	0.995	0.002
BEact (mmol/l)	0.072	0.123	0.013
BEstd (mmol/l)	0.856	0.016	0.041
O_2_ SAT (%)	0.969	<0.001	<0.001

pCO_2_, partial pressure of carbon dioxide (mmHg); pO_2_, partial pressure of oxygen (mmHg); ActHCO_3_, actual bicarbonate concentrations (mmol/l); StdHCO_3_, standard bicarbonate concentration (mmol/l); tCO_2_, total carbon dioxide (mmol/l); BEact, extracellular base excess (mmol/l); BEstd, blood base excess, (mmol/l);; O_2_ SAT, oxyhaemoglobin saturation (%).

## Discussion

Influences of storage time and temperature on blood gas and acid base indices has been studied before in man (Liss and Payne, 1993; Beaulieu *et al.*, 1999), in cattle (Szenci and Besser, 1990; Szenci *et al.*, 1991) and goat (Leal *et al.*, 2010).

In previous studies, it has been assumed that the blood gas and acid-base parameters usually stabilised in the blood samples between 15 min and 2 h in man (Mahoney *et al.*, 1991; Beaulieu *et al.*, 1999), up to 24 h in cattle (Szenci and Besser, 1990; Piccione *et al.*, 2004) and up to 6 h after refrigeration in goat (Leal *et al.*, 2010).

However, measurements of blood gas and acid-base analysis should be carried out as soon as possible after sampling (Piccione *et al.*, 2007; Cingi *et al.*, 2009). In the present study, the pH values of the blood samples kept at +4 ºC and 22-25 ºC were found to be suitable for clinical purposes up to 48 h and 12 h, respectively, while those stored at 37 ºC were found unsuitable for clinical usage (Fig. 1) (Table 3).

Furthermore, storage time was found to significantly alter the blood pH values in all groups except in group I, where storage time had no significant effect on pH value (*p* = 0.389). The prominent, rapid and significant decreases in blood pH (*p*<0.001) (Fig. 1) and BE (*p*<0.001) (Table 3) were observed for those samples stored at 37 ºC, compared to other groups (Groups I and II).

*In vitro*, metabolic process remains active during storage. Therefore such a drop in blood pH and BE could be attributed to the continuity of metabolism in the form of oxygen consumption as a result of anaerobic metabolism with generation of carbon dioxide in the tricarboxylic acid cycles (TCA) (Boink *et al.*, 1991) or accumulation of lactic acid due to glycolysis (Szenci and Besser, 1990).

Furthermore, the much greater drop in the pH of blood samples stored at 37 ºC may be attributed to lactic acid generation due to anaerobic metabolism as a result of high storage temperature (Liss and Payne, 1993). In the present study, increased values of HCO_3_ in all groups between 1 and 6 h may be due to increased values of tCO_2_ (George, 1986). Previously, Carlson and Bruss (2008) stated that HCO_3_ actually accounts for approximately 95 % of the measured tCO_2_. The later drop in concentrations of HCO_3_ could be attributed to accumulation of lactic acid due to glycolysis (Gokce *et al.*, 2004) or lowered tCO_2_ (George, 1986).

In the current study, increases in the pCO_2_ values of blood samples in groups I and II between 1 and 6 h, later lowered to values lower than their baseline values. Previous studies have reported that *in vitro* blood pCO_2_ values rises depending on aerobic and anaerobic metabolic activities in blood cells (Sandhagen *et al.*, 1988; Beaulieu *et al.*, 1999; Leal *et al.*, 2006). Furthermore, unlike O_2_, CO_2_ does not influx through plastic syringe wall (Mahoney *et al.*, 1991); therefore, the significant rises of pCO_2_ especially in groups I and II may be the result of high cell metabolic activities (Foster and Terry, 1967). Increased concentrations of pCO_2_ during an aerobic storage of bovine blood at +4 ºC have been documented (Poulsen and Surynek, 1977).

In the present study, pO_2_ values of the three groups showed significant increase in all time periods except in group III, pO_2_ concentrations decreased after 24 h (Table 1). Increased pO_2_ concentrations may be due to increased diffusion of oxygen through the plastic wall of the syringe (Paerregaard *et al.*, 1987; Beaulieu *et al.*, 1999) and to release of O_2_ from haemoglobin as a result of the reduction in blood pH (Szenci and Besser, 1990).

White blood cells are responsible for most of the aerobic metabolism in the blood (Liss and Payne, 1993; Kee *et al.*, 2010) causing consumption of oxygen in blood samples stored under *in vitro* anaerobic condition (Szenci and Besser, 1990). Furthermore, pO_2_ decreases in blood samples with anaemia (Haskins, 1977) and in samples with high white blood cell counts (Schmidt and Muller-Plathe, 1992). Since in the current study, the initial mean WBCs and RBCs counts and Hb concentrations of the blood samples were within normal reference range, decreased pO_2_ values after 24 h in blood samples stored at 37 ºC in comparison with their baseline value could be attributed to an increase in aerobic metabolism and O_2_ consumption by white blood cells (Liss and Payne, 1993).

In the present study, pO_2_ values were found lower in blood samples stored at 37 °C than those stored at +4 and 22-25 ºC (*p*<0.05). This finding supports the fact that aerobic metabolism of white blood cells was higher at 37 ºC than at RT and +4 ºC.

Increased tCO_2_ values in different groups between 1 and 6 h may be due to dissociations of H_2_CO_3_ and carbamino acids (George, 1986). Furthermore, O_2_ SAT values were decreased in association with the drop in blood pH in blood samples stored at 37 ºC. This could be attributed to shifting of haemoglobin-oxygen dissociation curve to the right (Bohr effect) as a result of decrease in blood pH (Szenci and Besser, 1990; Carlson and Bruss, 2008).

A time - group interaction on all measured parameters indicating the effects of storage temperature were not similar across the time periods. In the present study, it seemed that venous blood samples stored at different temperature regimens may be unsuitable for accurate blood gas and acid-base balance interpretation. In contrast, Leal *et al*. (2006) concluded that ovine venous blood samples stored at refrigeration are viable for blood gas and acid-base balance since the 24^th^ hours after collection. Such difference may be due to different species of sheep.

## Conclusion

In conclusion, blood gas and acid-base indices of blood samples stored at 22-25 (RT) and 37 ºC changed greatly when compared with the samples stored at +4 ºC (refrigeration). Blood gas and acid-base values of blood samples stored at 37 ºC were unstable, while acid-base indices of blood samples kept at 22-25 ºCseemed stable for up to 6 h. Furthermore, blood samples stored for more than 6 h at different temperature regimens were unsuitable for accurate interpretation of blood gas and acid-base indices in Baladi sheep.

### Clinical relevance

It is a fact that blood gas analysis and acid-base indices have a valuable diagnostic importance in ovine medicine. They are used diagnostically in cases of ruminal acidosis, pregnancy toxaemia, diarrhoea and renal insufficiency. Acid-base indices of ovine venous blood samples stored at 22-25 ºC (RT) or +4 ºC (refrigeration or ice box) remain within normal reference range and could be used diagnostically for clinical purposes up to six hours.

### Conflict of interest statement

None of the authors has any financial or personal relationships that could inappropriately influence or bias the content of the paper.

## References

[ref1] Beaulieu M, Lapointe Y, Vinet B (1999). Stability of pO_2_, pCO_2_ and pH in fresh blood samples stored in a plastic syringe with low heparin in relation to various blood-gas and haematological parameters. Clin. Biochem.

[ref2] Boink A.B, Buckley B.M, Christiansen T.F, Covington A.K, Maas A.H, Muller-Plathe O, Sachs C, Siggaard-Andersen O (1991). Recommendation on sampling, transport and storage for the determination of the concentration of ionized calcium in whole blood, plasma and serum. Ann. Biol. Clin.

[ref3] Carlson G.P, Smith B.P (1996). Clinical chemistry tests (acid–base imbalance). Large Animal Internal Medicine.

[ref4] Carlson G.P, Bruss M, Kaneko J.J, Harvey J.W, Bruss M.L (2008). Fluid, electrolytes, and acid-base balance. Clinical biochemistry of domestic animals.

[ref5] Cingi C.C, Civelek T, Acar A, Eryilmaz H (2009). Changes in blood gas composition and acid-base equilibriums in cattle blood samples kept under different temperature regimens and times. J. Anim. Vet. Adv.

[ref6] Coles E.H (1980). Veterinary Clinical Pathology.

[ref7] Duncan J.R, Prasse K.W (1986). Veterinary Laboratory Medicine Clinical Pathology.

[ref8] Dunn O.J, Clark V.A (2009). Basic Statistics: A Primer for the Biomedical Sciences.

[ref9] Foster J.M, Terry M.L (1967). Studies on the energy metabolism of human leucocytes. I. Oxidative phosphorylation by human leucocyte mitochondria. Blood.

[ref10] George J.W, Duncan J.R, Prasse K.W (1986). Water, electrolytes, and acid-base. Veterinary Laboratory Medicine.

[ref11] Gokce G, Citil M, Gunes V, Atalan G (2004). Effect of time delay and storage temperature on blood gas and acid-base values of bovine venous blood. Res. Vet. Sci.

[ref12] Haskins S.C (1977). Sampling and storage of blood for pH and blood gas analysis. J. Am. Vet. Med. Assoc.

[ref13] Kaneko J.J, Harvey J.W, Bruss M.L (2008). Clinical biochemistry of domestic animals.

[ref14] Kee J.L, Paulanka B.J, Polek C (2010). Handbook of fluid, electrolytes, and acid-base imbalances.

[ref15] Knowles T.P, Mullin R.A, Hunter J.A, Douce F.H (2006). Effects of syrine material, sample storage time and temperature on blood gases and oxygen saturation in arterialized human blood samples. Respir. Care.

[ref16] Leal M.L, Soares P.C, Beragnon H.G, Silva P.E, Ortolani E.L, Benesi F.J (2006). Effect of refrigeration on the hemogasometric examination of venous blood in sheep. Braz. J. Vet. Res. Anim. Sci.

[ref17] Leal M.L, Soares P.C, Cyrillo F.C, Benesi F.J (2010). Influence of refrigeration on blood gas analysis of caprine venous blood. Braz. J. Vet. Res. Anim. Sci.

[ref18] Liss H.P, Payne C.P (1993). Stability of blood gases in ice and at room temperature. Chest.

[ref19] Mahoney J.J, Harvey J.A, Wong R.J, Van Kessel A.L (1991). Changes in oxygen measurements when whole blood is stored in iced plastic or glass syringes. Clin. Chem.

[ref20] Paerregaard A, Nickelsen C.N, Brandi L, Andersen G.E (1987). The influence of sampling site and time upon umbilical cord blood acid–base status and pO_2_ in the newborn infant. J. Perinat. Med.

[ref21] Piccione G, Bertolluci C, Grasso F, Giudici E (2007). Changes in gas composition and acid-base values of venous blood samples stored under different conditions in 4 domestic species. Vet. Clin.Pathol.

[ref22] Piccione G, Caola G, Mortola J.P (2004). Day/night pattern of arterial blood gases in the cow. Respir. Physiol. Neurobiol.

[ref23] Poulsen J.S.D, Surynek J (1977). Acid–base status of cattle blood. Nord. Vet. Med.

[ref24] Pugh D.G (2002). Sheep and Goat Medicine.

[ref25] Radostits O.M, Gay C.C, Hinchcliff K.W, Constable P.D (2006). Veterinary Medicine: A textbook of the diseases of cattle, sheep, goat, pigs and horses.

[ref26] Sandhagen B, Hogman C.F, de Verdier C.H, Eriksson L (1988). Distrubution of blood gases, glucose and lactate within stored blood units. Vox.Sang.

[ref27] Schmidt C, Muller-Plathe O (1992). Stability of pO_2_, pCO_2_ and pH in heparinized whole blood samples: Influence of storage temperature with regard to leucocyte count and syringe material. Eur. J. Clin. Chem. Clin. Biochem.

[ref28] Szenci O, Besser T (1990). Changes in blood gas and acid–base values of bovine venous blood during storage. J. Am. Vet. Med. Assoc.

[ref29] Szenci O, Brydl E, Bajcsy C.A (1991). Effect of storage on measurement of ionized calcium and acid–base variables in equine, bovine, ovine, and canine venous blood. J. Am. Vet. Med. Assoc.

